# Roles Played by Stress-Induced Pathways in Driving Ethnic Heterogeneity for Inflammatory Skin Diseases

**DOI:** 10.3389/fimmu.2022.845655

**Published:** 2022-04-28

**Authors:** Taylor A. Jamerson, Qinmengge Li, Sutharzan Sreeskandarajan, Irina V. Budunova, Zhi He, Jian Kang, Johann E. Gudjonsson, Matthew T. Patrick, Lam C. Tsoi

**Affiliations:** ^1^ Department of Dermatology, Michigan Medicine, University of Michigan, Ann Arbor, MI, United States; ^2^ Department of Biostatistics, School of Public Health, University of Michigan, Ann Arbor, MI, United States; ^3^ Department of Dermatology, Northwestern Medicine, Northwestern University, Chicago, IL, United States; ^4^ Department of Urology, Northwestern Medicine, Northwestern University, Chicago, IL, United States; ^5^ Department of Computational Medicine and Bioinformatics, Michigan Medicine, University of Michigan, Ann Arbor, MI, United States

**Keywords:** African America, psoriasis, atopic dermatitis, stress, minority

## Abstract

Immune-mediated skin conditions (IMSCs) are a diverse group of autoimmune diseases associated with significant disease burden. Atopic dermatitis and psoriasis are among the most common IMSCs in the United States and have disproportionate impact on racial and ethnic minorities. African American patients are more likely to develop atopic dermatitis compared to their European American counterparts; and despite lower prevalence of psoriasis among this group, African American patients can suffer from more extensive disease involvement, significant post-inflammatory changes, and a decreased quality of life. While recent studies have been focused on understanding the heterogeneity underlying disease mechanisms and genetic factors at play, little emphasis has been put on the effect of psychosocial or psychological stress on immune pathways, and how these factors contribute to differences in clinical severity, prevalence, and treatment response across ethnic groups. In this review, we explore the heterogeneity of atopic dermatitis and psoriasis between African American and European American patients by summarizing epidemiological studies, addressing potential molecular and environmental factors, with a focus on the intersection between stress and inflammatory pathways.

## Introduction

Over 84 million Americans are impacted by at least one skin disease (1), posing significant health and economic burden. Atopic dermatitis (AD) and psoriasis are among the most common and widely studied immune-mediated skin conditions (IMSCs). With recent advances in genomic technology, studies over the past decade (2–8) have focused on elucidating the underlying mechanisms of disease pathogenesis for AD and psoriasis. More specifically, these studies have explored the genetic and molecular factors associated with the disease pathophysiology. However, the primary racial makeup of these studies has been predominantly European American (EA). While AD and psoriasis affect populations of different origins, their burden is exacerbated in some ethnic minority groups (9–11). Yet, there is a paucity of molecular studies describing the driving factors behind ethnic heterogeneity in the severity, presentation, and predominance of IMSCs.

Environmental factors, such as stress, are known to play roles in shaping the onset and clinical severity of IMSCs (12–14). While chronic stressors may impact any individual, unique psychosocial factors such as racism, discrimination, and acculturative stress (anxiety or tension related to efforts to adapt to the values of dominant culture within a society) are unique among ethnic minorities (15). Studies from the US Department of Health and Human Services Office of Minority Health show that African American (AA) adults living below the poverty line are twice as likely to experience psychological distress (16) and the downstream effects of these stressors can result in decreased likelihood of receiving medical care (17, 18). It is therefore important to understand the influence of stressors, including psychosocial stress, on the pathogenesis of IMSCs, especially as this may play role in the ethnic differences seen across common IMSCs.

In this review, we explore the heterogeneity in AD and psoriasis across AA and EA patients by summarizing epidemiological studies, as well as the potential molecular and environmental factors involved in disease pathogenesis. We also place particular focus on the intersections between known stress pathways and IMSC inflammatory pathways in the literature.

## Atopic Dermatitis

### Epidemiology

Atopic dermatitis (AD) is an inflammatory skin condition affecting more than 18 million adults in the United States (19, 20). This condition classically presents with pruritic, erythematous plaques involving the flexor surfaces, particularly in the antecubital and popliteal fossae. In Fitzpatrick skin types IV through VI however, eczematous patches often appear brown, purple, or ashen grey in color (11). Clinically, AD often presents with greater involvement of the flexural surfaces in adults, however patients of African descent are more likely to present with more prominent involvement of the extensor surfaces (21). Previous epidemiological studies using self-reported ethnic information highlight a slightly higher prevalence of AD in AA patients when compared with EAs (19.3% versus 16.1%) (22). This predominance is also seen at young age, with AA children found to be 1.7 times more likely to develop AD compared to their EA counterparts, even after adjusting for health insurance and socioeconomic status (11). In addition to reported racial differences in AD prevalence, AA children as a group has been reported to have more severe disease than EA children (23); the study also suggested structural racism or the increased proportion of AA children living in lower income, segregated communities with exposure to greater environmental stressors, are associated with disease severity. To further validate and understand the epidemiological factors involved, we studied the demographical variables from an insurance-claim database, Optum Clinformatics Data Mart (CDM), consisting of 1,458,417 AD patient records across 2014 to 2018 (**Table 1**). As expected, the association of AD-related clinical visits was significantly stronger at younger age (<18 years) for all ethnic groups compared with our reference age group (18-65 years) (OR:1.60, 1.95, 2.38, 1.92 for EA, Hispanic, Asian and AA populations, respectively). The older patient population group (>65 years) also had significantly stronger association with AD clinical visits (OR=2.18, 1.76, 1.52, 1.75 for the same four ethnic groups, respectively). The data importantly highlights that gender factors had the largest effect sizes in AA (e.g. OR=1.43 for female) compared to the other ethnic groups (OR between 1.23-1.35 for female). Patients with higher income are also associated with higher prevalance of clinical visit for AD.

**Table 1:** Risk factors for atopic dermatitis and psoriasis stratified by different ethnic groups.

		Atopic Dermatitis				Psoriasis			
		Odds Ratio	95% CI Lower bound	95% CI Upper bound	Significance	Odds Ratio	95% CI Lower bound	95% CI Upper bound	Significance
**Obesity**	**EA**	*1.49*	*1.48*	*1.51*	***	*1.89*	*1.86*	*1.91*	***
	**Hispanic**	*1.47*	*1.44*	*1.51*	***	*1.91*	*1.84*	*1.97*	***
	**Asian**	*1.32*	*1.25*	*1.40*	***	*1.98*	*1.83*	*2.14*	***
	**AA**	*1.55*	*1.51*	*1.59*	***	*1.75*	*1.68*	*1.81*	***
**Age (18-65 as ref)**	**EA (y)**	*1.60*	*1.58*	*1.62*	***	*0.18*	*0.17*	*0.18*	***
	**EA (o)**	*2.18*	*2.16*	*2.21*	***	*1.80*	*1.77*	*1.82*	***
	**Hispanic (y)**	*1.95*	*1.88*	*2.03*	***	*0.20*	*0.18*	*0.22*	***
	**Hispanic (o)**	*1.76*	*1.69*	*1.84*	***	*1.45*	*1.37*	*1.54*	***
	**Asian (y)**	*2.38*	*2.31*	*2.46*	***	*0.22*	*0.19*	*0.24*	***
	**Asian (o)**	*1.52*	*1.48*	*1.56*	***	*2.17*	*2.10*	*2.26*	***
	**AA (y)**	*1.92*	*1.87*	*1.97*	***	*0.21*	*0.20*	*0.23*	***
	**AA (o)**	*1.75*	*1.70*	*1.79*	***	*1.57*	*1.52*	*1.63*	***
**Gender (M as ref)**	**EA**	*1.35*	*1.34*	*1.36*	***	*1.10*	*1.09*	*1.11*	***
	**Hispanic**	*1.34*	*1.31*	*1.36*	***	*0.97*	*0.95*	*1.00*	.
	**Asian**	*1.23*	*1.19*	*1.27*	***	*0.84*	*0.80*	*0.89*	***
	**AA**	*1.43*	*1.40*	*1.46*	***	*1.14*	*1.10*	*1.18*	***
**$40K-$49K**	**EA**	*1.08*	*1.06*	*1.10*	***	*1.02*	*0.99*	*1.05*	.
	**Hispanic**	*1.05*	*1.01*	*1.09*	**	*1.00*	*0.94*	*1.06*	.
	**Asian**	*1.03*	*0.95*	*1.12*	.	*1.11*	*0.97*	*1.27*	.
	**AA**	*1.11*	*1.07*	*1.15*	***	*0.98*	*0.92*	*1.04*	.
**$50K-$59K**	**EA**	*1.13*	*1.11*	*1.15*	***	*1.07*	*1.05*	*1.10*	***
	**Hispanic**	*1.11*	*1.07*	*1.15*	***	*1.07*	*1.01*	*1.14*	*
	**Asian**	*1.09*	*1.01*	*1.18*	*	*1.14*	*1.01*	*1.30*	*
	**AA**	*1.22*	*1.18*	*1.27*	***	*1.00*	*0.94*	*1.06*	
**$60K-$74K**	**EA**	*1.21*	*1.19*	*1.23*	***	*1.14*	*1.11*	*1.16*	***
	**Hispanic**	*1.18*	*1.14*	*1.22*	***	*1.15*	*1.09*	*1.21*	***
	**Asian**	*1.09*	*1.02*	*1.17*	**	*1.13*	*1.01*	*1.25*	*
	**AA**	*1.32*	*1.27*	*1.37*	***	*1.11*	*1.05*	*1.18*	***
**$75K-$99K**	**EA**	*1.31*	*1.29*	*1.33*	***	*1.23*	*1.20*	*1.25*	***
	**Hispanic**	*1.31*	*1.27*	*1.35*	***	*1.25*	*1.19*	*1.31*	***
	**Asian**	*1.13*	*1.06*	*1.20*	***	*1.24*	*1.13*	*1.37*	***
	**AA**	*1.37*	*1.32*	*1.42*	***	*1.26*	*1.19*	*1.33*	***
**$100K+**	**EA**	*1.71*	*1.69*	*1.73*	***	*1.52*	*1.49*	*1.55*	***
	**Hispanic**	*1.61*	*1.57*	*1.65*	***	*1.62*	*1.55*	*1.69*	***
	**Asian**	*1.25*	*1.19*	*1.32*	***	*1.37*	*1.26*	*1.49*	***
	**AA**	*1.63*	*1.58*	*1.68*	***	*1.56*	*1.48*	*1.65*	***

The demographical variables from an insurance-claim database (CDM) of 1,458,417 atopic dermatitis and 272,913 psoriatic patient records were analyzed across 2014 to 2018. For age, individuals of 18-65 years were used as reference for <18 years age (y) and >65 years age (o) groups; for socio-economic status, individuals with household income <$40,000 were used as reference. For significance level: *0.01<p ≤ 0.05, **0.001<p ≤ 0.01, ***p ≤ 0.001. Income is reported in the United States dollar (USD).

Optum Clinformatics Data Mart (CDM) is a claim-based Electronic Health Record (EHR) database containing demographic, diagnosis, pharmacy, and lab analyte records for > 63 million de-identified patients from 2001 to 2018 in US (https://www.optum.com/business/solutions/life-sciences/real-world-data/claims-data.html). We restricted our study to only consider recent patient visit between 2014-2018, and implemented a case-control study framework to further decrease the size of patients without the target disease. In the analysis, we used all the AD or psoriasis patients as our case sample, and randomly drew 3 million patients from the rest of the data and included only the patients that visited and filed claims between 2014-2018. We then fitted logistic regressions on the AD or psoriasis indicator adjusting for obesity, age, gender, household income and race. Obesity, age, gender, household income covariates are coded by the reference cell coding scheme, with reference levels to be non-obesity, 18-65, male and below $40,000 respectively.

### Genetics

While the cause of AD is complex, studies over the last decade have provided insights into genetic and environmental factors associated with disease pathogenesis and severity. Filaggrin (*FLG*), a protein encoding gene from the epidermal differentiation complex (EDC) and expressed in the keratinized layer of the epidermis, plays an important role in skin barrier function, for example by promoting keratinocyte differentiation and rapid cell death (24). FLG expression is immune-modulated by both aryl hydrocarbon receptor signaling and cytokines, resulting in dysregulation within the lesional skin (25). The locus harboring *FLG* has been identified as one of the strongest genetic signals associated with AD (26). Carriers of the *FLG* loss-of-function (LOF) variants have increased odds (3-fold) of having AD. While LOF *FLG* mutations are risk factors for the development of AD in patients of European and Asian descent, this association has not been reported among individuals of African descent. In fact, loss of function *FLG* mutations are thought to be less common in AA patients with AD when compared to EAs (27), and a recent study suggests that the common *FLG* mutation found in AA patients are distinct from those in EA and Asians (28). Nevertheless, decreased expression and mutations of filaggrin-2 (*FLG2*), also from EDC, have been associated with persistent symptoms of AD in AA, while such variations are absent or infrequently found in AD patients of European ancestry (29). The first genome-wide significant association at rs3811419 for AD in AA patients was found to be an expression quantitative trait loci (eQTL) for *THEM4* (a gene associated with allergy), and *FLG-AS1* (a non-coding RNA that overlaps the filaggrin gene) in blood. The association between the risk allele of this eQTL locus and increased expression of *FLG-AS1* can potentially offer an alternative mechanism explaining skin barrier deficiency in AA, although further research is required (30).

While AA patients are more likely to present with more severe AD than EAs patients (22), a recent study challenged the perception that racial heterogeneity in the severity of AD is genetically driven (31). This study found that observed differences of AD in AA patients, including disease severity, were not associated with a continuous measure of African genetic ancestry. This finding supports epidemiologic, rather than genetic, associations with disease severity and suggests the potential roles of other factors such as environmental components (e.g., stress, pollution, social determinants of health) in disease heterogeneity and their influence on disease pathogenesis.

### Stress and Immunologic Parameters

Though most studies describing the molecular signature of AD have been conducted in patients of European ancestry, a recent study by Wongvibulsin et al. confirmed previously reported Th_2_/Th_22_ skewing in AA patients with AD, along with upregulated Th_1_ cytokines in lesional skin of AD, contribute to the increased disease severity in AA patients (32). The authors also reported elevated serum C-reactive protein (CRP), ferritin, and blood eosinophils in AA patients when compared to EA patients with AD. In addition, Schmeer et al. measured serum CRP levels across ethnic groups in children aged 2 to 10 years and found significantly higher serum measurements in AA and Hispanic children when compared to EA children (33). In adults, increased activity of two essential pro-inflammatory transcription control pathways, NF*ĸ*B and AP-1, was found in AA subjects who also independently reported experiencing greater perceived racial discrimination as assessed through a 17-item Perceived Ethnic Discrimination Questionnaire—Community Version (34) when compared to EA subjects. These findings suggest that psychosocial stressors may play a role in the increased activation of pro-inflammatory pathways.

Existing studies have proposed several mechanisms in the intersection of psychological stress and AD. The first involves an association between the hypothalamus-pituitary-adrenal (HPA) axis and AD (35). AD has been linked to HPA axis alterations, which contribute to several downstream pathological changes in the skin. Adrenocorticotropic hormone (ACTH), which promotes glucocorticoid secretion, can create a negative feedback loop in the HPA axis in response to stress, and early life adversity can modulate the regulation of HPA axis through epigenetic modification of the glucocorticoid receptors in the skin (36). An important modulator in the HPA axis and AD pathogenesis is IL-18, a member of the IL-1 cytokine family implicated in various immune-mediated skin disease including AD, psoriasis, alopecia areata, dermatomyositis, and cutaneous lupus erythematous (37–41). ACTH can also activates caspase-1 and keratin 1, leading to keratinocyte production of IL-18 (42). In the absence of IL-12, IL-18 has been shown to modulate the Th_2_ pathway, inducing expression of IL-4, IL-13, and IgE by basophils (43) The downstream effect of these expressed factors has been linked to AD pathogenesis. IL-13 is a prominent Th2 cytokine (6, 44); and serum IgE levels are elevated among AA patients with AD when compared to all other racial/ethnic groups (22). Lastly, IL-18 is thought to directly activate mast cells, leading to release of the enzyme chymase which cleaves pro-IL-18 and potentially accelerates the inflammatory response in AD lesions (45). Existing evidence shows higher clinical AD severity index score (SCORing Atopic Dermatitis or SCORAD) correlate with increased serum IL-18 concentration in AD patients (46). However, as far as the authors are aware, no studies have compared HPA modulation and IL-18 expression across different ethnic groups in AD patients.

Another possible mechanism for the intersection of stress with AD pathogenesis involves chronic psychological stressors that induce serum epinephrine, norepinephrine, and cortisol levels, triggering a shift to a Th_2_ cytokine profile (47). Though this has not been substantiated in AD through measurement of serum levels, salivary cortisol level has been correlated with SCORAD index scores among patients with elevated stress levels (48). In addition, genetic variants of interferon regulatory factor 2 (IRF2), a protein with crucial roles in immune response, including the regulation of IFNγ and basophil expansion, as well as the transcription of gasdermin D, are associated with AD risk in both AA and EAs (49). Gasdermin D is a critical mediator of inflammatory pathologies, with its non-canonical inflammasome signaling pathway leading to proteolytic activation of IL-1B and IL-18. The pro-inflammatory cytokine, IL-1B, along with TNF-alpha induce expression of 11 beta-hydroxysteriod dehydrogenase, are critical enzymes involved in cortisol synthesis and hypothesized to modulate pro-inflammatory cytokine expression in keratinocytes (50). Our recent work using skin and 3D human skin equivalents (HSE) also demonstrates that skin from AA patients exhibits stronger inflammatory response when compared with that from EA patients. The differentially expressed genes (DEG) in AA skin include those that encode immunoglobulins and their receptors such as *FCER1G*; proinflammatory genes such as *TNF*, *IL-32*; and different EDC and keratin genes. By investigating the effect of TNF signaling on HSE, we further demonstrated enhanced TNF pro-inflammatory effects in AA HSE (51).

## Psoriasis

### Epidemiology

Psoriasis is a chronic IMSC with variable prevalence across populations. It has a lower prevalence among AA patients when compared with EAs (0.22% to 1.9% in AA vs 1.28% to 3.6% in EA) in the United States (52). Nevertheless, AA patients have been found to have more extensive disease involvement and higher rate of comorbidities including diabetes, hypertension, and hyperlipidemia, after controlling for age and body mass index, when directly compared to EAs (53, 54). While erythematous plaques with thick overlying scale is characteristic of plaque psoriasis, AA patients often present with less conspicuous erythema and a higher degree of dyspigmentation (55), which can often take months to years to resolve. These pigmentary changes can often be of equal or greater concern to patients than the psoriasis itself and contributes to the report of increased disease severity, greater psychological impact, treatment dissatisfaction, and decreased quality of life among AA patients (9, 54). There is currently little consensus regarding gender differences in psoriasis (56), particularly for underrepresented ethnic groups.

We used the CDM insurance-claim database to review the associated demographic factors with 272,913 psoriatic patients with diagnosis between 2014 and 2018 (**Table 1**). Specifically, the impact of gender on psoriasis was found to be significantly different across ethnic groups, with EA and AA women having higher psoriasis diagnosis rates compared to men (OR=1.10, 1.14); in contrast, the gender effect was not significant in Hispanic patients (p=0.098), and within the Asian population, females had significantly lower rate (OR=0.84). Previous studies showed ambiguous results when estimating the prevalence of psoriasis in each gender, but overall the differences are very minimal (57). We also observed that psoriasis risk was significantly higher for people with obesity across different ethnic groups, and the association between clinical visits for psoriasis was significantly lower among patients with lower income.

### Genetics

Like AD, psoriasis has a complex genetic architecture with >80 different disease susceptibility loci identified, the majority of which only contribute to a modest effect of disease association. The HLA-Cw6 is the most prominent disease-associated signal, with >4 OR being revealed (58), and multiple different work have also highlighted other independent signals in the MHC region (59, 60). Despite >10 years since the first GWAS for psoriasis was conducted, large-scale genetic studies for psoriasis have been exclusively been based on EA, Chinese, and Japanese populations (4, 7, 61–64). Up to now, only very limited small GWAS study on psoriasis is based on African ancestry (65). While this can be attributed by the lower incidence rate of psoriasis and the more complex design for GWAS in individuals of African ancestry, the lack of diversity in genetic research for psoriasis needs to be addressed in order to understand the disease heterogeneity and to facilitate the fine-mapping of ethnic-shared/unique causal variations.

### Stress and Immunologic Parameters

Increased corticotropin-releasing hormone (CRH) from stress leads to increased serum cortisol levels and decreased brain derived neurotrophic factor (BDNF) (66). CRH, encoded by the *CRHR-1* gene, is a peptide hormone that is essential in the physiologic response to stress. Elevation in serum CRH levels is associated with exposure to stress in psoriatic and AD patients, providing a link between stress and the HPA in both conditions (67). In psoriatic patients, increased serum CRH and decreased skin CRHR-1 expression have also been linked to the induction of vascular endothelial growth factor (VEGF) release from mast cells (67). VEGF is a known growth factor involved in the pathogenesis of psoriatic lesions and it is therefore hypothesized that these pathways are linked through increased levels of CRH playing a role in the activation of mast cells that release VEGF (68). CRH has also been implicated in stimulating the production of IL-6 and IL-11, and the downregulation of IL-1B, IL-2, and IL-18 in keratinocytes (69, 70); and a previous work has found elevated serum cortisol level in psoriatic patients when compared to healthy controls under increased psychosocial stress (71). These authors proposed that psoriatic patients have a robust neuroendocrine response in the presence of acute stressors, increasing vulnerability to psoriatic activity. On the other hand, localized glucocorticoid deficiency in psoriatic skin is associated with epidermal differentiation and inflammatory response, and restoring glucocorticoid biosynthesis can normalize these processes (72). Topical glucocorticoid has been shown to be responded by AA skin in a stronger degree, with the response of AA skin associating with inflammation and metabolic disruptions while the genes responding to glucocorticoid in EA are associated with cell barrier modifications (73). Animal studies have demonstrated the reduction of brain-derived neurotrophic factor (BDNF), a protein with skin related functions in humans including the induction of apoptosis in basal keratinocytes, in response to acute stress (66, 74, 75). These findings suggest that psychosocial stress as a potential factor linking decreased BDNF levels in psoriatic patients. Nevertheless, there is still very limited study that describes the differential impact of stress on chemokine or cytokine expression among AA and EA patients with psoriasis, which requires attention and future investigations.

## Conclusion

AD and psoriasis are two of the most common IMSCs that can have significant impact on the quality of life in patients, especially in racial/ethnic minorities. Individuals of African ancestry are more likely to develop AD in childhood, experience more severe disease, and can have atypical presentation in adulthood with greater involvement of the extensor surfaces. Though the prevalence of psoriasis is lower among AA patients, they can suffer from more extensive disease involvement, experience significant post-inflammatory changes, and report decreased quality of life. Previous studies have attempted to account for the differences in AD or psoriasis disease severity and prevalence through variable demographic and genetic components; however, as highlighted in a recent review, the differential severity of AD between AA and EA patients cannot solely be explained by association with genetic ancestry (31). Research on the ethnic heterogeneity of IMSCs should place more focus on the role of psychosocial stressors on inflammatory cytokine and chemokine expression and overall disease pathogenesis of these conditions. We review existing studies examining the role of psychological or psychosocial stress at the molecular level in AD and psoriasis, highlighting the role of the HPA axis and IL-18 in AD, CRH and BDNF in psoriasis, and cortisol levels in both.

## Author Contributions

LT and TJ planned and designed the work. QL, SS, and MP conducted the EHR analysis. All authors contributed to the article and approved the submitted version.

## Funding

This work was supported by the National Psoriasis Foundation (LT, MP, and JG), and awards from the National Institutes of Health (K01AR072129 to LT; 1P30AR075043 to LT, MP, and JG). MP, LT, TJ were also supported by the Dermatology Foundation.

## Conflict of Interest

JG has served as a consultant to AbbVie, Eli Lilly, Almirall, Celgene, BMS, Janssen, Prometheus, TimberPharma, Galderma, Novatis, MiRagen, AnaptysBio and has received research support from AbbVie, SunPharma, Eli Lilly, Kyowa Kirin, Almirall, Celgene, BMS, Janssen, Prometheus, and TimberPharma. LT has received support from Janssen and Galderma.

The remaining authors declare that the research was conducted in the absence of any commercial or financial relationships that could be construed as a potential conflict of interest.

## Publisher’s Note

All claims expressed in this article are solely those of the authors and do not necessarily represent those of their affiliated organizations, or those of the publisher, the editors and the reviewers. Any product that may be evaluated in this article, or claim that may be made by its manufacturer, is not guaranteed or endorsed by the publisher.
